# Effect of medial arch-heel support in inserts on reducing ankle eversion: a biomechanics study

**DOI:** 10.1186/1749-799X-3-7

**Published:** 2008-02-20

**Authors:** Daniel TP Fong, Mak-Ham Lam, Miko LM Lao, Chad WN Chan, Patrick SH Yung, Kwai-Yau Fung, Pauline PY Lui, Kai-Ming Chan

**Affiliations:** 1 Department of Orthopaedics and Traumatology, Prince of Wales Hospital, Faculty of Medicine, The Chinese University of Hong Kong, Hong Kong, China; 2 The Hong Kong Jockey Club Sports Medicine and Health Sciences Centre, Faculty of Medicine, The Chinese University of Hong Kong, Hong Kong, China; 3 Gait Laboratory, Department of Orthopaedics and Traumatology, Alice Ho Miu Ling Nethersole Hospital, Hong Kong, China

## Abstract

**Background:**

Excessive pronation (or eversion) at ankle joint in heel-toe running correlated with lower extremity overuse injuries. Orthotics and inserts are often prescribed to limit the pronation range to tackle the problem. Previous studies revealed that the effect is product-specific. This study investigated the effect of medial arch-heel support in inserts on reducing ankle eversion in standing, walking and running.

**Methods:**

Thirteen pronators and 13 normal subjects participated in standing, walking and running trials in each of the following conditions: (1) barefoot, and shod condition with insert with (2) no, (3) low, (4) medium, and (5) high medial arch-heel support. Motions were captured and processed by an eight-camera motion capture system. Maximum ankle eversion was calculated by incorporating the raw coordinates of 15 anatomical positions to a self-compiled Matlab program with kinematics equations. Analysis of variance with repeated measures with post-hoc Tukey pairwise comparisons was performed on the data among the five walking conditions and the five running conditions separately.

**Results:**

Results showed that the inserts with medial arch-heel support were effective in dynamics trials but not static trials. In walking, they successfully reduced the maximum eversion by 2.1 degrees in normal subjects and by 2.5–3.0 degrees in pronators. In running, the insert with low medial arch support significantly reduced maximum eversion angle by 3.6 and 3.1 degrees in normal subjects and pronators respectively.

**Conclusion:**

Medial arch-heel support in inserts is effective in reducing ankle eversion in walking and running, but not in standing. In walking, there is a trend to bring the over-pronated feet of the pronators back to the normal eversion range. In running, it shows an effect to restore normal eversion range in 84% of the pronators.

## Background

Excessive pronation (or eversion in frontal plane) at ankle joint during repetitive impact in heel-toe running correlates with lower extremity overuse injuries and musculoskeletal pathologies, such as patellofemoral joint syndrome [1]. Ankle pronation (and its opposite, supination) refers to the calcaneal motion with respect to the talus orientation at the subtalar joint. In heel-toe walking and running, ankle pronation is accompanied by knee flexion and internal tibial rotation. At heel strike, pronation of subtalar joint unlocks the midtarsal joints and allows the foot to absorb shock and adapt to uneven terrains. In take off, subtalar joint supinates and relocks the midtarsal joints, which turns the foot into a rigid lever for push-off [2]. The axis orientation of the subtalar joint is about 42 and 23 degrees to the human anatomical transverse plane and sagittal plane respectively [3]. Since the axis does not coincide with the human anatomical reference frame, the subtalar joint movement is often described as a tri-planar motion. The motion in frontal plane is often termed calcaneal or heel inversion/eversion [4], which describes the foot segment rotation about the anterior-posterior axis [5].

Moulded foot orthoses have been shown to be successful in treating such injuries and reducing the symptoms [6] by realigning the foot anatomy, controlling excessive pronation and reducing internal tibial rotation [7]. Numerous prophylactic or therapeutic devices, such as motion control shoe, orthoses, orthotic devices, inserts and others, have emerged to limit the pronation range during running. In evaluating the effect of these devices to control pronation during running, orthopaedics and biomechanics researchers often investigate the rearfoot kinematics, or to be specific, the calcaneal motion in respect to the talus bone. Previous researches showed that the effects are still unclear [8]. Scherer [9] showed that orthotic inserts are useful in relieving heel and plantar fascilitis pain, however, Gross and co-workers [10] showed no improvement or even increased symptom severity in runners being prescribed with orthotics. Moreover, there are many types of commercially available orthotic in the marker, including half insert or full insert, with different degree of support in medial and lateral arch-heel regions [3,11,12] Therefore, the effect of orthotic inserts is product-specific, thus, biomechanics evaluation of orthotic inserts is necessary before the inserts are introduced to the market.

This study aims to evaluate the effect of orthotic inserts with different degree of medial arch-heel support in reducing maximum ankle eversion in standing, walking and running.

## Methods

Twenty six children subjects (age = 6.9 ± 1.0 yrs, height = 1.16 ± 0.05 m, mass = 20.9 ± 3.7 kg, male = 15, female = 11) were recruited in this study. All subjects were right-legged, and were able to walk independently. Exclusion criteria were the present of serious foot problems, lower limb or back fractures in the past one year, balancing problems, unequal leg length, and high medial foot arch, as examined by an orthopaedic specialist. Written informed consent was collected from parent of each subject before the test. The university ethics committee approved the study.

**Figure 1:**
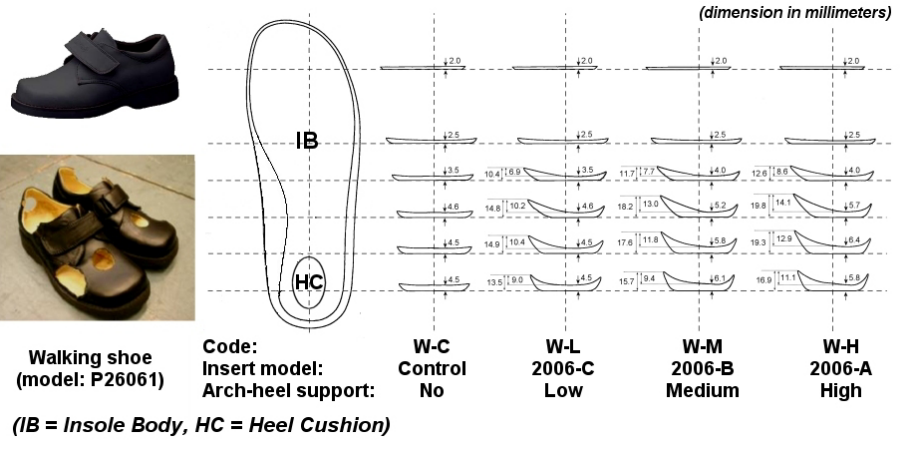
The walking shoe and the corresponding set of inserts.

**Figure 2:**
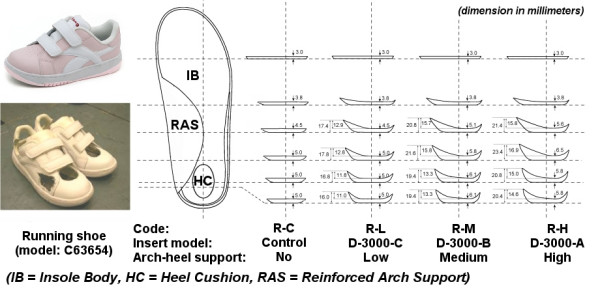
The running shoe and the corresponding set of inserts.

The test was conducted in the Gait Laboratory in the Department of Orthopaedics and Traumatology at the Alice Ho Miu Ling Nethersole Hospital. Each subject performed walking (Code = W) and running (Code = R) trials in each of the following conditions: (1) barefoot (Code = BF), and shod condition with insert with (2) no (Code = C), (3) low (Code = L), (4) medium (Code = M), and (5) high (Code = H) medial arch-heel support. Two different series of inserts were used, i.e. W series for walking shoe and R series for running shoes, as they are with difference in dimension, shape, material and reinforced arch support to fit in the shoes (Figure 1 and 2). Walking shoe (Dr Kong Footcare Limited, Model: P26061) and running shoe (Dr Kong Footcare Limited, Model: C63654) of size EUR 29 were used for walking and running trials respectively. To facilitate locating the markers, holes were cut on the shoes to allow the markers to be seen from outside. For each subject, the shoes were fastened by a research assistant to be as tight as possible without introducing discomfort to the subject. For condition of no medial arch-heel support, a flat insert was used as control. The details of shoe and insert model of the ten testing conditions is shown in Table 1.

**Table 1:** Details of shoe and insert model of the ten testing conditions.

Condition/Medial arch-heel support	Code	Shoe	Insert	Material/Stiffness (Young's Modulus, 10^6^N/m^2^)		
				IB	HC	RAS
Walking						
Barefoot	W-BF	-	-	-	-	-
Shod/No	W-C	P26061	Control	-	-	-
Shod/Low	W-L	P26061	2006-C/I98008	PU 50/0.45	Poron 15/0.66	-
Shod/Medium	W-M	P26061	2006-B/I97007	PU 50/0.45	Poron 15/0.66	-
Shod/High	W-H	P26061	2006-A/I96006	PU 70/0.55	Poron 15/0.66	-
Running						
Barefoot	R-BF	-	-	-	-	-
Shod/No	R-C	C63654	Control	-	-	-
Shod/Low	R-L	C63654	D-3000-C/I3030	PU 30/0.30	Poron 15/0.66	TPU 98/1.15
Shod/Medium	R-M	C63654	D-3000-B/I3031	PU 30/0.30	Poron 15/0.66	TPU 98/1.15
Shod/High	R-H	C63654	D-3000-A/I3032	PU 30/0.30	Poron 15/0.66	TPU 98/1.15

(Shoes and inserts were from Dr Kong Footcare Limited. IB = Insole Body, Hardness value in Shore D Scale; HC = Heel Cushion, Poron density value in lb/ft^3^, RAS = Reinforced Arch Support, hardness value in Shore A Scale)

**Figure 3:**
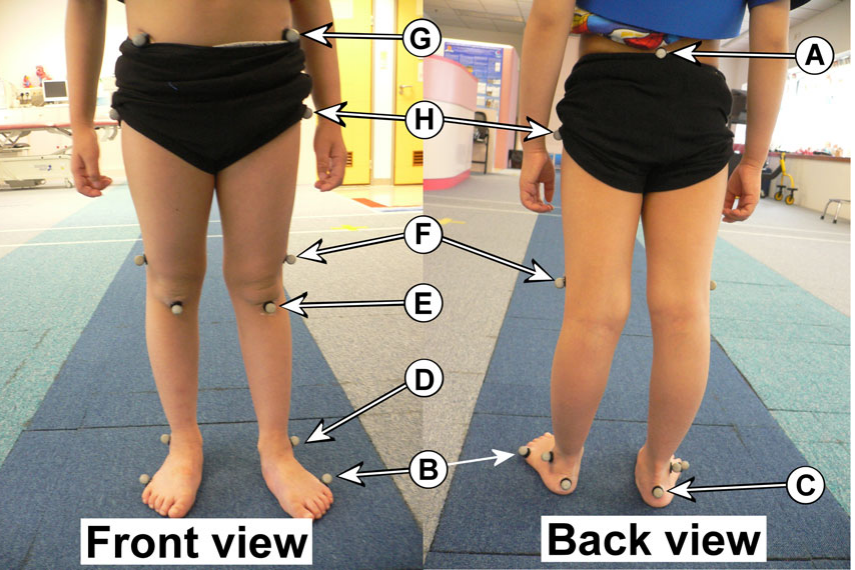
The reflective markers attached on the major anatomical landmarks.

Before the test, each subject's lower extremity anthropometric data was measured. The subject was then requested to wear tight shorts and shirts in order to expose the major anatomical landmarks for attaching reflective skin markers. Fifteen markers were attached to the sacrum (A), bilateral fifth metatarsal head (B), calcaneus (C), lateral malleolus (D), tibial tubercle (E), lateral femoral epicondyle (F), anterior superior iliac spine (G) and greater trochanter (H) (Figure 3), following the Helen Hayes model [13].

In order to show the ankle orientation during standing, walking and running, we define an "offset position" as the reference for comparison. The offset neutral position of the subject was determined by a physiotherapist. The foot was off the floor, and the talo-navicular joint was palpated to be in a maximally congruent position, that is, the head of the talus was not palpable medially or laterally when both sides of the joint were simultaneously palpated just anterior to the medial and lateral mallleoli [14]. The offset neutral position was captured by an eight-camera motion capture system (Vicon, UK) at 120 Hz. Each subject was then instructed to stand still in anatomical position, i.e. an erect upright standing posture, in the middle of the walking path. The lower extremity orientation was captured by the motion capture system in order to determine the ankle eversion angle. The static trial was performed in all conditions.

Subjects were requested to perform heel-toe walking and running at 1.30 and 1.66 m/s respectively. The achieved speed was obtained from the motion capture system immediately after each trial, and was reported to the subject for adjusting their speed. Practice trials were allowed until the subject could perform the motion at the required speed. Each subject performed three trials per each of the ten conditions on a 15-meter path in a randomized sequence. Successful trials were defined when the subject stepped on a force plate (AMTI, USA) in the middle of the walking path with the right foot. The vertical ground reaction force data was used to determine the stance phase, which was defined when the force exceeded 10N (about 5% of the subject's body weight). Raw coordinates of the 15 markers during the stance phase was trimmed and extracted. A self-compiled Matlab program was used to calculate the ankle kinematics with the equations suggested by Vaughan and co-workers [15]. Ankle eversion was defined as the internal rotation of the foot segment from the offset neutral position (A negative value means an inverted orientation). In static trial, the average ankle eversion angle was obtained. In walking and running trials, the maximum ankle eversion angle during the stance phase was obtained.

From the barefoot static trial, each subject was identified to be a pronator if the ankle eversion angle exceeded 13 degrees [16], or a normal subject if the angle did not exceed the limit. Chi-square and independent t-tests were conducted to determine any difference among the demographics of the two groups. If no significant was found, repeated measures analysis of variance (ANOVA) with repeated measures would be conducted for statistical analysis, otherwise, repeated measures analysis of covariance (ANCOVA) with repeated measures would be conducted, with the demographic variables showing difference as the covariates. Statistical analysis (either ANOVA or ANCOVA with repeated measures) was conducted separately in each of the pronators and normal subject group, on (1) static trial with walking inserts (W series), (2) static trial with running inserts (R series), (3) walking trial with walking inserts (W series), and (4) running trial with running inserts (R series). When significant effect was determined, post-hoc Tukey pairwise comparisons were conducted to determine if the shod conditions differ from barefoot condition, and if the inserts with medial arch-heel support differ from the insert with no support. Statistical significance was set at 0.05 level.

## Results

Thirteen subjects were identified as pronators as they had eversion angle exceeding 13 degrees in static barefoot trial (mean = 16.1 ± 2.1 degrees, range = 13.1–19.8 degrees). The other 13 subjects were classifies as normal subjects (mean = 7.4 ± 3.0 degrees, range = 2.9–11.7 degrees). The demographics were shown in Table 2. Chi-square test showed no difference in the male to female ratio between two groups. Independent t-tests showed the only difference is the ankle eversion angle in barefoot static trial (p < 0.05), but not in age, height and mass. Therefore, ANOVA was performed for statistical analysis.

**Table 2:** Demographics of the two groups and the results of statistical tests.

	Pronator (N = 13)	Normal (N = 13)	chi-square^a^/t-test^b ^results
Male/Female	6/7	9/4	No significant difference^a^
Age (years)	7.0 ± 0.9	6.8 ± 1.1	No significant difference^b^
Height (m)	1.17 ± 0.05	1.15 ± 0.06	No significant difference^b^
Mass (kg)	21.0 ± 3.4	20.9 ± 4.2	No significant difference^b^
Eversion (deg)	16.1 ± 2.1	7.4 ± 3.0	p < 0.05^b^

### Static trial with walking shoe and walking inserts (W series)

**Figure 4:**
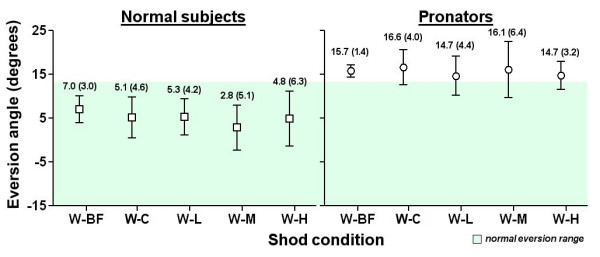
Results of static trial with walking shoe and walking inserts (W series).

The eversion angle of each condition was shown in Figure 4. For normal subjects, the shod condition with flat insert (W-C) slightly decreased the eversion angle from 7.0 to 5.1 degrees, but the effect was not significant. Insert with low, medium and high medial arch-heel support did not show any significant effect with the condition with flat insert (W-C). The eversion angles of all conditions fell within the normal eversion range (within 13 degrees). For pronators, the W-C condition showed a small but insignificant increase of eversion angle, from 15.7 to 16.6 degrees. All other inserts did not show any effect. All eversion angles were out of the normal eversion range.

### Static trial with running shoe and running inserts (R series)

**Figure 5:**
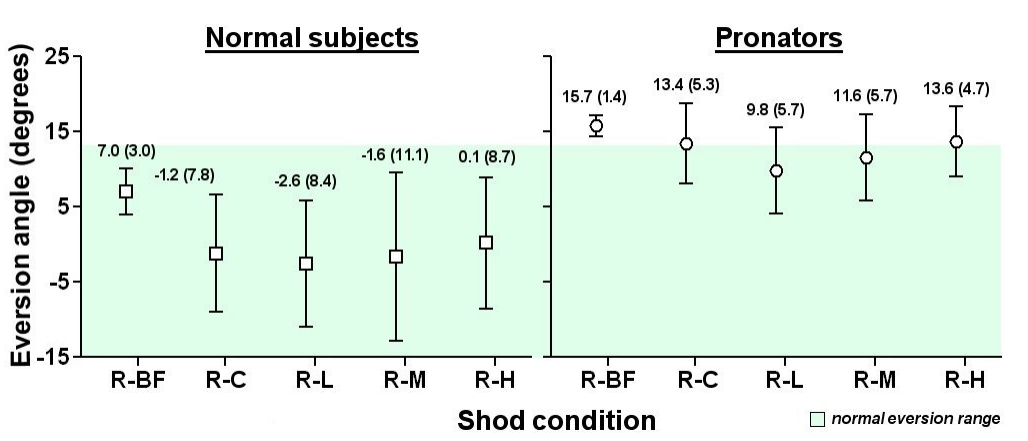
Results of static trial with running shoe and running inserts (R series).

The eversion angle of each condition was shown in Figure 5. For normal subjects, the shod condition with flat insert (R-C) slightly decreased the eversion angle from 7.0 to -1.2 degrees (a negative sign means an inverted ankle orientation). The effect was not significant. Insert with low, medium and high medial arch-heel support did not show any significant effect with the condition with flat insert (R-C). The eversion angles of all conditions fell within the normal eversion range. For pronators, the R-C condition showed a small but insignificant decrease of eversion angle, from 15.7 to 13.4 degrees. All other inserts did not show any effect, however, R-L and R-M fell within the normal eversion range and R-H was just out of the range.

### Walking trial with walking shoe and walking inserts (W series)

**Figure 6:**
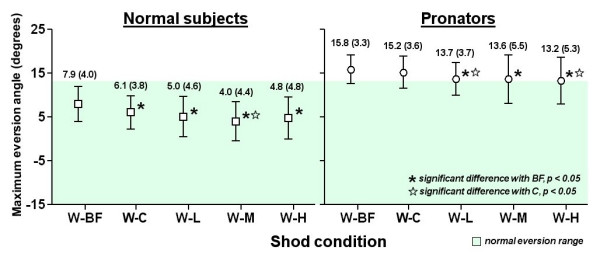
Results of walking trial with walking shoe and walking inserts (W series).

The eversion angle of each condition was shown in Figure 6. For normal subjects, the shod condition with flat insert (W-C) significantly decreased the maximum eversion angle from 7.0 to 6.1 degrees (p < 0.05). In addition, all other inserts showed significant reduction of maximum eversion (p < 0.05). When compared with W-C condition, insert with medium medial arch-heel support further reduced the maximum eversion from 6.1 to 4.0 degrees (p < 0.05). The eversion angles of all conditions fell within the normal eversion range. For pronators, the W-C condition showed a small but insignificant decrease of eversion angle, from 15.7 to 15.2 degrees. All other inserts showed significant reduction with the barefoot condition (W-BF) to 13.2–13.7 degrees (p < 0.05). When compared with W-C condition, W-L and W-H showed additional effect (p < 0.05). All eversion angles were slightly out of the normal eversion range.

### Running trial with running shoe and running inserts (R series)

**Figure 7:**
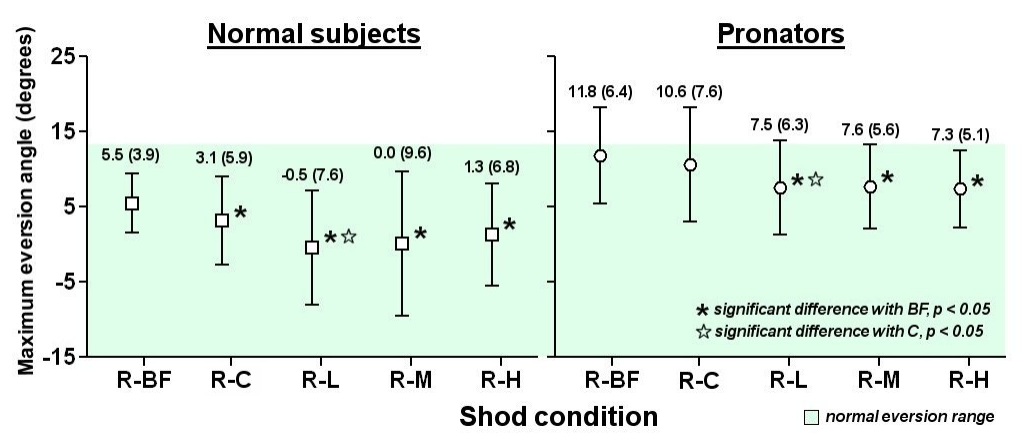
Results of running trial with running shoe and running inserts (R series).

The eversion angle of each condition was shown in Figure 7. For normal subjects, the shod condition with flat insert (R-C) significantly decreased the maximum eversion angle from 5.5 to 3.1 degrees (p < 0.05). In addition, all other inserts showed significant reduction of maximum eversion (p < 0.05). When compared with R-C condition, insert with low medial arch-heel support further reduced the maximum eversion from 3.1 to -0.5 degrees (p < 0.05). The eversion angles of all conditions fell within the normal eversion range. For pronators, the R-C condition showed a small but insignificant decrease of eversion angle, from 11.8 to 10.6 degrees. All other inserts showed significant reduction with the barefoot condition (R-BF) to 7.3–7.6 degrees (p < 0.05). When compared with R-C condition, R-L showed additional effect (p < 0.05). All eversion angles were within the normal eversion range.

## Discussion

Significant effects were found in dynamic trials (walking and running) but not in static trials (standing). This may be due to the nature of the motion. In standing, both feet support the human body, in a symmetric way. Therefore, the lack of medial support of the right foot could be somewhat compensated by the support of the left foot, and vice versa. In dynamic trial, the maximum eversion angles were obtained during the single-leg stance phase of right foot. In this period of time, the left foot was in swing phase and could not provide any support to the body. Therefore, the right foot alone had to support the full body weight in walking, and even 2–3 times of the body weight in running. In such situation the medial arch-heel support become more demanding, and thus the effect of inserts were found significant in dynamic trials. Therefore, evaluation of inserts should be done in dynamics trials to demonstrate the effect in dynamic situation.

For walking trials, the insert with medium medial arch-heel support (W-M) was found to be effective when compared to the insert with no support (W-C) in normal subjects. It showed a 2.1 degrees reduction of maximum eversion angle. The insert with low (W-L) and high (W-H) support were found effective in pronators. They reduced the maximum eversion angle by 1.5 and 2.0 degrees respectively. When compared to barefoot condition, all inserts with the walking shoe showed significant reduction of maximum eversion angle for both normal subjects (2.9–3.9 degrees) and pronators (2.1–2.6 degrees). For pronators, the W-L, W-M and W-H conditions showed a trend to bring the over-pronated ankle back to the normal eversion range, which is within 13 degrees. However, all three conditions recorded a mean maximum eversion angle slightly greater than 13 degrees.

For running trials, the insert with low support (W-L) was effective to insert with no support (W-C) for both normal subjects and pronators. Again, all three inserts with medial support showed significant reduction of maximum eversion angle when compared to barefoot condition. In normal subjects, the inserts were successful to limit ankle eversion, as the maximum eversion angle almost equaled the neutral offset position. In pronators, although all conditions were within the normal eversion range, the R-L, R-M and R-H showed that the range of 1 SD among the mean value still lied within the normal eversion range. This indicated that 84% of the pronators would have a maximum eversion angle within the normal range.

## Conclusion

The inserts with medial arch-heel support were found to be effective in reducing maximum eversion angle in dynamic trials but not static trials. In walking, the inserts successfully reduced the maximum eversion angle by 2.1 degrees in normal subjects and by 1.5–2.0 degrees in pronators. The inserts showed a trend to bring the over-pronated feet of pronators back to the normal eversion range. In running, the insert with low medial arch-heel support significantly reduced maximum eversion angle by 3.6 and 3.1 degrees in normal subjects and pronators respectively. The inserts successfully restored normal eversion in 84% of the pronators.

## Competing interests

Research funds were received from Dr Kong Footcare Limited in support of this work.

## Authors' contributions

DTPF designed the study, conducted statistical analysis and drafted the manuscript. MHL, MLML and CWNC conducted the data collection and data processing. PSHY and KYF designed the study, provided laboratory equipments and interpreted the data. PPYL designed the study, interpreted the data and critically revised the manuscript. KMC conceived and coordinated the study. All authors read and approved the final manuscript.
